# Long-term evaluation (10 years) of the outcomes of Herbst functional appliance in pharyngeal dimensions and hyoid bone position

**DOI:** 10.1590/2177-6709.30.3.e252543.oar

**Published:** 2025-10-20

**Authors:** Thagid Yasmin Leal ALMEIDA, Tiago FIALHO, Karina Maria Salvatore de FREITAS, José Fernando Castanha HENRIQUES, Marcos Roberto de FREITAS

**Affiliations:** 1 São Paulo University, Bauru Dental School, Department of Orthodontics (Bauru/SP, Brazil).; 2 Ingá University Center, Department of Orthodontics (Maringá/PR, Brazil).

**Keywords:** Malocclusion, Angle Class II, Orthodontic appliances, functional, Pharyngeal airway, Hyoid bone, Effects, Long-term, Má oclusão Classe II de Angle, Aparelhos ortodônticos funcionais, Região branquial, Osso hioide, Tempo

## Abstract

**Objective:**

This study aimed to evaluate the long-term changes in the pharyngeal dimensions and hyoid bone position in Class II malocclusion patients treated with Herbst functional appliance.

**Material and Methods:**

The sample comprised 15 skeletal Class II malocclusion patients (13.00 ± 1.21 years) treated with Herbst functional appliance and followed for a mean period of 10 years (10.73 ± 3.67 years). Lateral headfilms were used to evaluate the pharyngeal dimensions and hyoid bone position, and the measurements were performed with Dolphin Imaging 11.9. Intragroup comparison between the evaluation stages was performed with repeated measures ANOVA, followed by Tukey tests if necessary. Results were considered statistically significant at p<0.05.

**Results:**

Regarding the pharyngeal cephalometric variables, only the hipo pharynx space increased significantly in the treatment period, and all of them remained stable remained stable during follow-up period. The hyoid bone moved significantly forward and downward during the treatment period and even further forward during the long-term posttreatment period.

**Conclusion:**

The outcomes of Herbst functional appliance in pharyngeal dimensions and hyoid bone position are stable over the years.

## RESUMO

**Objetivo:**

Este estudo teve como objetivo avaliar as alterações a longo prazo nas dimensões faríngeas e na posição do osso hioide em pacientes com má oclusão de Classe II tratados com aparelho funcional Herbst.

**Material e Métodos:**

A amostra foi composta por 15 pacientes com má oclusão esquelética de Classe II (13,00 anos ± 1,21) tratados com aparelho funcional Herbst e acompanhados por um período médio de 10 anos (10,73 anos ± 3,67). Radiografias laterais da cabeça foram utilizadas para avaliar as dimensões faríngeas e a posição do osso hioide, e as medidas foram realizadas com o programa Dolphin Imaging 11.9. A comparação intragrupos entre os estágios de avaliação foi realizada com o teste ANOVA de medidas repetidas, seguida do teste de Tukey, se necessário. Os resultados foram considerados estatisticamente significativos em p < 0,05.

**Resultados:**

Em relação às variáveis cefalométricas faríngeas, apenas o espaço hipofaríngeo aumentou significativamente no período de tratamento, e todos eles permaneceram estáveis durante o período de acompanhamento. O osso hioide moveu-se significativamente para frente e para baixo durante o período de tratamento e ainda mais para frente durante o período de pós-tratamento de longo prazo.

**Conclusão:**

Os resultados do aparelho funcional Herbst nas dimensões faríngeas e na posição do osso hioide são estáveis ao longo dos anos.

## INTRODUCTION

Class II malocclusion is one of the most prevalent orthodontic problems globally and represents approximately one-third of the patients seeking orthodontic treatment.^1^ This malocclusion is present in about 15% of the United States population,^1^ and 38% of Brazilian children.^2^

The relationship between sagittal skeletal pattern and airway space has been the subject of several studies, and the different anteroposterior skeletal patterns influence airway dimensions.^3^ ^,^ ^4^ Compared to children with normal occlusions, children with skeletal Class II malocclusion have significantly smaller nasopharyngeal dimensions^5^ and have a higher risk of future respiratory problems.^4^

Functional Appliances (FAs) are routinely used to treat children with Class II skeletal malocclusion due to mandibular retrognathism,^6^ and it may help increase the airway dimensions, preventing respiratory disorders.^7^ Thus, in some cases, orthodontic treatment can be chosen that improves not only the maxillomandibular relationship but also reduces the risk of acquiring Obstructive Sleep Apnea Syndrome (OSAS).^8^

The Herbst appliance is a fixed functional orthopedic device for treating Class II malocclusion with several advantages compared to removable functional appliances.^9^ Among its advantages, it may lead to changes in facial profile and muscle activity.^10^ Many variations in Herbst design have occurred over the years. The Cantilever Bite Jumper (CBJ) marked a significant advancement in Herbst appliance designs, particularly in terms of facilitating dentition transition.^11^

Previous studies have evaluated the association between fixed functional orthopedic appliances to treat Class II patients with mandibular retrognathism and changes in airway dimensions,^12^ ^,^ ^13^ and it is a controversial subject. Specifically, about the association between airway and Class II malocclusion treatment with Herbst functional appliance, some studies showed dimension improvement of specific regions of the upper airway^14^ ^-^ ^17^ while others showed no significant changes in any of them.^13^ ^,^ ^18^ Furthermore, it is important to realize that some studies of airway dimensions changes by Class II malocclusion treatment with Herbst appliance associated this protocol with rapid maxillary expansion (RME).^14^ ^,^ ^16^ Modification of pharyngeal airway by RME is described in the literature,^19^ and these results must be carefully evaluated.

Long-term follow-up of any orthodontic treatment is essential to assess the effectiveness of the treatment. Therefore, this study aimed to evaluate long-term changes in airway dimensions and hyoid bone position in patients with Class II malocclusion treated with the Herbst functional appliance. 

## MATERIAL AND METHODS

The present study was approved by the Ethics Committee in Human Research of *Faculdade de Odontologia de Bauru, Universidade de São Paulo* (FOB-USP, Brazil), under protocol number CAAE: 22082219.0.0000.5417.

The sample size was calculated with a statistical power of 0.80 and an alpha of 5% to detect a mean difference of 0.25 mm for the middle airway space (U-MPW) with a standard deviation of 0.32 mm, obtained in the study conducted by Göymen, Mourad, and Güleç.^12^ The result showed the need for 15 patients. 

The sample for the study group was taken retrospectively from the Department of Orthodontics, FOB-USP, and the patients selected had attended by graduate students. The inclusion criteria were patients aged between 11 and 15 years old, with initial Angle Class II, division 1 malocclusion and mandibular retrognathism; who underwent functional orthopedic treatment with Herbst, associated with fixed orthodontic appliance; ANB angle > 4º; SNB angle < 80º; complete orthodontic records, including lateral headfilms available in three moments: T1, pretreatment; T2, post-treatment (Herbst followed by fixed orthodontic appliance); and T3, long-term follow-up (at least seven years post-treatment). All patients underwent a mouth breathing assessment conducted by the orthodontist during their orthodontic treatment, and the findings were documented in their medical records. The exclusion criteria were history of previous orthodontic/orthopedic treatment; loss of any permanent teeth; palatal/lip cleft symptoms; chronic mouth breathing; permanent snoring; and tonsillectomy or adenoidectomy.

The patients selected were treated without extractions with a Herbst variation (Cantilever Bite Jumper, CBJ, Ormco Corporation, Orange, California, USA) orthopedic approach, followed by fixed appliances. After correction of Class II malocclusion, fixed orthodontic appliances were used to refine the occlusion (Roth prescription, Morelli, Sorocaba, Brazil). Patients wore a fixed mandibular retainer for at least five years after treatment and a Hawley maxillary plate for one year after treatment.

The lateral headfilms were digitized and analyzed with Dolphin Imaging 11.9 software (Patterson Dental Supply, Inc., Chatsworth, California, USA) by a single examiner (T.Y.). The software corrected the image magnification factors according to the respective X-ray unit’s settings and instructions.

**Figure 1:**
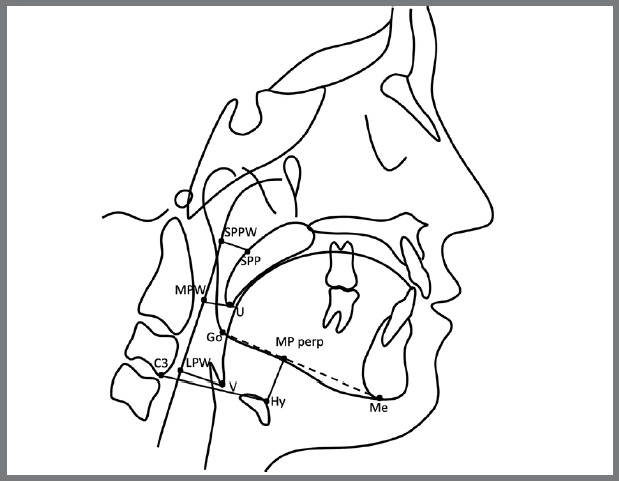
Diagram of landmarks and variables associated with upper airway and hyoid bone.

The cephalometric landmarks and analysis of the pharyngeal structures and hyoid bone position were based on the methods described previously by Liu et al.^20^ and Zong et al.^21^ (Table 1, Fig. 1). In addition, the cephalometric variables representing the dentoskeletal pattern were evaluated by Steiner analysis.^22^ A customized cephalometric analysis generated 13 variables, six angular and seven linear, for each tracing (Table 2).

**Table 1:** Definitions of the cephalometric landmarks used in the present study.

Cephalometric landmarks	Definition
N	The most anterior point of the frontonasal suture in the sagittal view
S	Center of the pituitary fossa
A	Point of maximum concavity in the midline of the alveolar process of the maxilla in the sagittal view
B	Point of maximum concavity in the midline of the alveolar process of the mandible in the sagittal view
Me	Most inferior point of the mandibular symphysis in the sagittal view
Go	The deepest point of the curvature of the angle of the mandible between the inferior border of corpus and posterior border of the ramus of mandible in sagittal view
U1	Maxillary incisor tip
L1	Mandibular incisor tip
U	The lower end of the soft palate
V	The most posteroinferior point on the base of the tongue
SPP	Point of intersection of a line from soft palate center perpendicular to the posterior pharyngeal wall and posterior margin of the soft palate
SPPW	Point of intersection of a line from soft palate center perpendicular to the posterior pharyngeal wall and posterior pharyngeal wall
MPW	Foot point of a perpendicular line from point U to the posterior pharyngeal wall
LPW	Foot point of a perpendicular line from point V to the posterior pharyngeal wall
Hy	The most anterior point on the hyoid bone
C3	The most anteroinferior point on the corpus of the third cervical vertebra

**Table 2:** Summary of the variables used for the cephalometric comparisons.

Variable	Interpretation
Skeletal component	
SNA	Angle between SN and NA
SNB	Angle between SN and NB
ANB	Angle between AN and NB
SN.GoGn	Angle between S-N and Go-Gn
Dentoalveolar component	
U1-NA	Distance between NA line and the most anterior point of the maxillary incisor crown
U1.NA	Angle between NA line and the long axis of the maxillary incisor
L1-NB	Distance between NB line and the most anterior point of the lower incisor crown
L1.NB	Angle between NB line and the long axis of the mandibular incisor
Pharyngeal airway	
SPP-SPPW	Superior airway space: Distance between SPP and SPPW (nasopharynx / palatopharynx)
U-MPW	Middle airway space: Distance between U and MPW
V-LPW (mm)	Inferior airway space: Distance between V and LPW
Hyoid bone position	
Hy-C3	Distance between the most anterior point of the hyoid bone and the most anteroinferior point in the body of the third cervical vertebra
Hy-MPperp	Distance from the mandibular plane (Go-Me) perpendicular to the hyoid bone

### ERROR STUDY

Thirty percent of the headfilms were randomly selected, retraced, and remeasured by the single examiner (T.Y.) after a month interval. IntraClass Correlation Coefficient (ICC) was used for test reliability.

### STATISTICAL ANALYSES

Normal distribution was evaluated and confirmed with Shapiro-Wilk tests. 

Intragroup comparison between the three evaluation stages was performed with repeated measures ANOVA, followed by Tukey tests if necessary. 

Results were considered statistically significant at P<0.05. Statistical analyses were performed with Statistica software (Statistica for Windows 12.0; Statsoft, Tulsa, Okla, USA). 

## RESULTS

The sample was composed of 15 subjects (5 female; 10 male), with a mean age of 13.00 ± 1.21 years. Herbst appliance was used during a mean period of 1.18 ± 0.19 years, and the second period of treatment (comprehensive treatment) lasted a mean time of 3.01 ± 1.48 years (Table 3). Post-treatment changes were evaluated 10.73 ± 3.67 years after orthodontic treatment.

**Table 3:** Descriptive characteristics of the sample.

X	n = 15	
Stage/Period	Mean	SD
T1 age	13.00	1.21
T2 age	18.02	1.78
T3 age	28.75	4.37
Total treatment period (T2-T1)	5.02	1.55
Long-term Posttreatment period (T3-T2)	10.73	3.67
Sex	Male	Female
	10 (66.66%)	5 (33.33%)

IntraClass correlation coefficients for the two separate measurements on the lateral headfilms ranged between 0.968 (Hy-Mp perp) and 0.997 (Md1.NB), indicating excellent reliability.^23^

Regarding treatment changes, the skeletal components showed significant increase in the SNB angle, improvement in the maxillomandibular relationship, and mandibular counterclockwise rotation. There were no significant changes in the dentoalveolar components. The lower airway, the distance between the hyoid bone and the third cervical vertebra and the distance between the mandibular plane perpendicular to the hyoid bone have undergone significant changes, increasing in the treatment period (Table 4).

**Table 4:** Intragroup comparisons of the cephalometric variables among the three stages (repeated measures ANOVA, followed by Tukey tests).

Variables	T1	T2	T3	p
	Mean (SD)	Mean (SD)	Mean (SD)	
Skeletal components				
SNA (degrees)	81.76 (2.66)	81.15 (2.99)	81.32 (3.14)	0.416
SNB (degrees)	76.22 (1.97)^A^	77.85 (2.23)^B^	78.32 (2.77)^B^	0.008*
ANB (degrees)	5.51 (1.77)^A^	3.29 (1.89)^B^	2.99 (1.95)^B^	0.000*
SN,GoGn (degrees)	31.54 (4.95)^A^	29.60 (3.77)^B^	29.04 (5.48)^B^	0.047*
Dentoalveolar components				
U1-NA (mm)	4.18 (3.02)	4.61 (1.64)	4.74 (1.86)	0.672
U1.NA (degrees)	23.03 (10.20)	25.32 (6.64)	25.95 (7.29)	0.380
L1-NB (mm)	4.64 (2.72)	6.07 (1.87)	5.74 (2.24)	0.066
L1.NB (degrees)	25.45 (8.48)	30.49 (5.81)	28.72 (5.63)	0.084
IMPA	92.9 (8.15)	95.4 (7.35)	96.1 (7.32)	0.206
Pharyngeal airway				
SPP-SPPW (mm)	11.68 (2.41)	12.23 (2.12)	10.99 (2.32)	0.104
U-MPW (mm)	9.88 (2.63)	9.43 (2.53)	9.62 (2.58)	0.758
V-LPW (mm)	15.94 (2.53)^A^	18.32 (3.60)^B^	19.09 (3.93)^B^	0.007*
Hyoid bone position				
Hy-C3 (mm)	31.45 (3.35)^A^	34.65 (4.88)^B^	36.48 (5.57)^C^	0.000*
Hy-MPperp (mm)	15.14 (3.29)^A^	18.58 (3.88)^B^	21.07 (4.22)^B^	0.002*

* Statistically significant at p<0.05.

Different superscript letters in the same row indicate the presence of a statistically significant difference between the stages.

In the long-term posttreatment period, only the distance between the hyoid bone and the third cervical vertebra suffered significant changes, increasing (Table 4).

## DISCUSSION

Although previous studies evaluated the association between airways and functional appliances,^3^ ^,^ ^24^ ^-^ ^26^ the long-term follow-up is important to discuss the effectiveness and stability of the treatment and remains unclear. Our study assessed upper airway dimensions and hyoid bone position changes in Class II patients with mandibular retrognathism treated with Herbst CBJ functional appliance for an average of 10 years (10.73 ± 3.67 years) of follow-up.

The sample size was sufficient to give reliability to the results. Statistical significance was achieved, indicating that the treatment effect was large enough to be detected in a limited sample. In addition, although a small sample size, it is substantial because the subjects were evaluated more than ten years post-treatment. Another study conducted similar research showed similar samples.^18^

For ethical reasons, it seems obvious the impossibility of long-term follow-up of untreated Class II individuals. Moreover, most of the growth of airway structures finishes in early childhood, and only a slight continuous increase of about 1 mm was detected between 6 and 17 years of age.^27^ Our sample was 18.02 ± 1.78 years old at the end of treatment, then there was expected to be no increase in the airway dimensions from growth in the follow-up period. 

Although three-dimensional imaging (3D) using cone-beam computed tomography (CBCT) to evaluate changes in airway dimensions is preferred to a lateral cephalometric radiograph, CBCT is not a standard diagnosis method in Orthodontics and routine use is not recommended for legal and ethical reasons because of radiation exposure.^28^ Furthermore, the literature shows a significant correlation between sagittal cephalometric measurements of the airway and 3D analysis with CBCT imaging.^29^ Therefore, this method for analyzing the airway is still a valid tool as it is inexpensive, has minimal dose radiation, and gives accurate measurements.^30^

Regarding the treatment changes in the skeletal components, the sample underwent an improvement in mandibular position, as previously reported for similar appliances, probably due to treatment and normal growth changes;^31^ mandibular counterclockwise rotation which is in agreement with a previous study,^16^ and improvement in maxillomandibular relationship, as the literature has already shown,^32^ represented mainly by the changes in the mandibular component and growth.^6^

There were no significant changes in the dentoalveolar variables with treatment and it is corroborated by previous studies that found that Herbst CBJ variation causes less mandibular incisor protrusion when compared to other designs of this appliance.^31^

Concerning the airway variables, a significant change was observed only in the lower airway space during treatment, increasing. This find is in agreement with previous study that found that the hypopharynx region has the greatest dimensional increase in treatment with the Herbst appliance,^15^ and other studies that reported increase in hypopharynx with Herbst treatment.^14^ ^,^ ^16^ ^,^ ^33^ This fact is probably related to the forward shift of the mandible, plus some growth effect.^15,16^ Some studies that evaluated the effects of the Herbst device on the airways reported an increase in the oropharyngeal region, but this was not found in our study.^14^ ^-^ ^17^ Dentoalveolar changes by functional orthopedic appliances, especially mandibular incisor protrusion, may be responsible for more anterior tongue position and increase in airways dimensions.^34^ In our study the sample did not show significant changes in the dentoalveolar variables, which can explain the insignificant changes in the oropharynx. 

Most studies that evaluated the relationship between treatment with Herbst appliance and the maxillary airway did not evaluate the hyoid bone position. Changes in hyoid bone position are related to mandibular position changes.^35^ In the present study hyoid bone was moved significantly forward and downward in the treatment period, which corroborates with previous studies that showed anterior displacement of the mandible by the functional appliances improving the horizontal position of the hyoid bone and, consequently, the position of the tongue, increasing upper airway dimensions.^14^ ^,^ ^25^ The downward displacement of the hyoid bone after Class II functional treatment was also reported in the literature previously.^36^ ^,^ ^37^ Change in the hyoid bone position in a superior direction was reported after functional advancement of the mandible, however, in the long term, it resumed its original position as a compensatory action.^38^

More information about the long-term effects of fixed functional appliances in the airway dimension is needed. To the best of our knowledge, only one study has evaluated the long-term effects of fixed functional appliances on upper airway,^18^ and they did not analyze the changes in the hypopharynx dimension and hyoid bone position, and included patients treated with extraction in their sample. Tooth extraction can interfere with airway dimensions, although in different ways in different malocclusions. In Class II patients, after extraction of maxillary premolars, there was a reduction in the dimensions of the velopharynx.^39^ In contrast, mesial movements of molars in Class I patients increased the posterior space of the tongue, consequently increasing the dimensions of the airway.^39^ Some authors have researched the long-term effects of removable functional appliances in the upper airway,^37^ ^,^ ^40^ however it is important to note that different functional appliances may affect the dentoalveolar and skeletal structures,^24^ as well in the upper airway and hyoid bone position.^25^ ^,^ ^41^

In the present study, only the horizontal position of the hyoid bone changed significantly in the long-term posttreatment period, moving forward, which agrees with the study by Ulusoy et al.^37^ All other variables remained stable during this period, converging with results from previous long-term follow-up studies with functional appliances.^37^ ^,^ ^40^

Our results have a relevant clinical impact, considering that Orthodontists can ensure that the outcomes of Herbst functional appliance in pharyngeal dimensions and hyoid bone position are stable over the years.

As this is a retrospective study, we do not know if special instruction was given to the patient about the tongue and head position in the lateral cephalogram records, affecting our findings. Another limitation of this study was that a retrospective study design could not include body mass index (BMI). Body mass index has been shown to impact the airway dimensions in children^42^ and adults^43^ and may have influenced the airway measurements. 

Future studies should be conducted to correlate dimensional changes in the airways and the impact on respiratory problems and sleep disorders.

## CONCLUSION

During treatment, the sample showed a significant increase in the hypopharynx, which may be related to the forward shift of the mandible, plus some growth effect. The hyoid bone was moved forward and downward as a treatment effect. All studied variables remained stable in long-term follow-up, except for the horizontal position of the hyoid bone which changed significantly, moving forward.

The results of this research demonstrate that Class II division 1 patients can benefit from the Herbst appliance not only for dental improvements, the primary objective of orthodontic treatment, but also for airway enlargement, particularly in the hypopharynx. This effect is especially significant in mouth breathing patients.
